# Changes in public satisfaction with GP services in Britain between 1998 and 2019: a repeated cross-sectional analysis of attitudinal data

**DOI:** 10.1186/s12875-022-01696-w

**Published:** 2022-04-18

**Authors:** Motab Aljohani, Michael Donnelly, Ciaran O’Neill

**Affiliations:** 1 Centre for Public Health, School of Medicine, Dentistry and Biomedical Sciences, Queen’s University Belfast, Belfast, Northern Ireland; 2 Public Health Department, College of Health Science, Saudi Electronic University, Riyadh, Saudi Arabia

**Keywords:** Health reforms, Health policy, Patient satisfaction, Primary care, GP services

## Abstract

**Background:**

Between 1998 and 2019, the structure and process of general practitioner services in Britain underwent a series of reforms and experienced distinct funding environments. This paper examines changes in satisfaction with GP services over time against this backdrop.

**Methods:**

Data were extracted from the British Social Attitudes Survey for the period 1998–2019. Logistic regression analyses investigated changes in overall satisfaction and among specific population sub-groups differentiated by socio-demographic characteristics whilst taking account of time trend and interaction effects between sub-group membership and time trend.

**Results:**

Sustained and significant changes in satisfaction coincided closely with changes to the funding environment. Distinct patterns were evident among sub-groups. Satisfaction appeared to fall more sharply during austerity for low income groups, older people and people who had fewer formal qualifications/years in education.

**Conclusion:**

While a series of policy initiatives were adopted over the period examined, public satisfaction seemed to move in a manner consistent with levels of government expenditure rather than exhibiting distinct breaks that coincided with policy initiatives. As services recover from the pandemic it will be necessary to invest in a significant and sustained way to rebuild public satisfaction.

**Supplementary Information:**

The online version contains supplementary material available at 10.1186/s12875-022-01696-w.

## Background

General practice is the first and commonly the most frequent point of contact between a health service and the public. The successful provision and delivery of primary care (to which general practice is central) is key to improving population health [1, 2], the efficient operation of the broader healthcare system [3–5] and the pursuit of health care equity objectives [6–8]. In this context, there is a need for researchers and policy makers to give analytical attention to the operation of general practice and the impact of changes to it on service users and the wider public. General practitioner services in the UK, are delivered free at the point of use to all citizens [9]. The arrangements under which care has been delivered and the levels of funding to support the delivery of care have, however, been the subject of frequent change [10]. These changes may reasonably be expected to impact on user experience, public perceptions, and the differential experience of sub-groups among the population insulated or exposed to differing degrees by their needs or access to other resources. Analysing the impact of such changes is not straightforward, however. Changes to provider behaviour may anticipate a policy change following its announcement but preceding its adoption. Equally, inertia in the system may see changes in behaviour lag behind the adoption of a policy while within a context of ongoing reform, multiple policies may not only overlap in their timing but may interact in their effects making it difficult to disentangle the role of any specific policy. More generally, the ability of a service to meet the expectations of the public will depend on levels of investment in the service as well as the policy context.

In Britain the election of the first New Labour government in 1997 ushered in a period of reform for general practice. In England, GP fundholding was abolished, and Primary Care Groups (which subsequently became Primary Care Trusts) changed the structures around general practice in 2002. Similar changes were adopted in Wales and Scotland in 2003 and 2004 respectively [9]. A new contract which applied across the UK was introduced in 2004 that changed the responsibilities of GPs as well as introducing an element of performance related pay under the Quality Outcomes Framework thus changing the process of care [11]. Further structural reforms followed in 2006 and 2013 and in 2019 another new contract was introduced [9, 11, 12]. The period 1997–2019 in other words can be seen as one of near perennial reform.

In terms of funding, the history of this period is somewhat less turbulent and can be divided broadly into three parts. With the exception of a slight pause in 2006/07, annual spending on public healthcare rose as a percentage of GDP in the UK steadily from 5.3% in 1997/98 to 8.26% in 2009. Between 2009/10 and 2018/19 this fell back to 7.73%, despite population growth and ageing, before commencing an upward trend again in 2019 to 7.98%. For England while the number of GPs per head of population increased throughout the period, this increase was not uniform, and indeed there was an actual fall in the full-time equivalent number of GPs in specific years. For example, while the number of GPs per head of population rose between 1997 and 2009 in the UK it fell between 2009 and 2018 [13].

A number of studies have examined the impact of policy and the broader economic context on satisfaction with health services [14–17] These have included studies that sought to examine the role of patient experience and health status [18]. Analytical investigations of changes in satisfaction with primary care services over time are by comparison relatively sparse [19–21] While various studies have sought to compare satisfaction between groups differentiated by gender, income, age and education [22, 23] few have sought to examine variation in the experiences of different groups over time with specific respect to general practice as the economic and/or policy context changed. The study by L’Esperance et al. [24] is a notable exception to this. Using data from service users, the study highlighted a clear correlation between patient experience and practice funding across a range of domains. It did not look specifically at satisfaction, nor the experience of the wider public and was confined to a relatively short period 2013–2017. In this paper, we examine changes in public satisfaction with NHS general practice over the period 1998–2019 in Britain. We compare the experience of groups differentiated by observable characteristics including age, income, ethnicity, education, jurisdiction and sex within a context of changing funding and policy environments.

## Methods

### Data

Data were extracted from the British Social Attitudes Survey (BSAS) for the period 1998 to 2019. BSAS is a repeated cross-sectional survey of attitudes undertaken annually in Britain. The survey is designed to yield a distinct representative sample of community dwelling adults aged 18 and over. A multistage sampling approach is used to construct the sample based on a representative selection of postcodes, random sampling of addresses within those postcodes and random sampling from among adults aged over 18 within the household [25]. While questions vary each year, depending on the themes to be explored in that year, core socio-demographic questions are repeated each year as are a range of attitudinal questions including (of interest here) those related to satisfaction with various publicly funded health services. Individuals are recruited to different versions of the questionnaire in the interests of minimising respondent fatigue but the sample as noted is weighted to remain representative. Respondents are asked to rate their satisfaction with general practitioner services on a five-point scale that ranged from very satisfied, through satisfied, neither satisfied nor dissatisfied to dissatisfied and very dissatisfied. Options to report “don’t know” are also provided. Satisfaction was re-defined as a dichotomous variable in which very satisfied, satisfied and neither satisfied nor dissatisfied were coded as one and other levels of satisfaction defined as zero. Other values were treated as missing. Respondents are asked the question whether or not they have used services. While others have used ordered logistic regression to analyse satisfaction [14] in other contexts we deliberately chose to use logistic regression following the analysis by others of satisfaction with health services using this same data source [26]. The use of logistic regression facilitates comparisons with other relevant studies and, arguably, allows a more intuitive presentation of results.

A range of socio-demographic characteristics were also extracted from the survey. These included age (whether respondent was 18 to 64 or 65 or over), household income (in quartiles), ethnicity (whether or not a respondent was White) education (whether a respondent had obtained a degree); sex (male/female), marital status (whether or not a respondent was married/living as such) and whether the household included dependent children. In each case the comparator group was defined as the base category (eg aged less than 65 and lowest quartile of income). The use of socio-demographic data that were extracted was informed by previous analyses of satisfaction [27, 28]. As data constituted a repeated cross-sectional survey, sampling weights for each year were extracted as well as the year in which the survey was conducted. The number of GPs per head of population was higher in Scotland compared to England and Wales throughout the period under examination [29] and, so, whether a respondent resided in Scotland or another part of Britain was also extracted. Ethical approval was not required as our study involved secondary analysis of an anonymised publicly available dataset.

### Analysis

Data were pooled across the 21 years of annual surveys. Descriptive statistics (proportions together with their associated 95% confidence intervals) were used to profile the data. Multivariable logistic regression analyses were undertaken in which satisfaction with GP services was estimated in relation to socio-demographic characteristics together with a trend variable for year of survey. Analyses were repeated to investigate covariates that interacted with the trend variable to explore the possibility that estimated coefficients may not remain stable over time – for example, that the relationship between age (say) and satisfaction may vary over time. Results were reported as odds ratios with predicted margins ‘graphed’ to facilitate presentation of results. All analyses were applied to weighted data and then repeated for unweighted data. Analyses were conducted in Stata version 16.0. We did not define a structural break in the time series or examine the data by, for example, conducting an interrupted time series given the rolling nature of reforms during this period and contemporaneous economic changes. Instead, a descriptive approach was adopted in which we examined changes to predicted satisfaction over time, differences in this indicator between specific groups and public sector pending on health as a percentage of GDP. The strengths and limitations of our approach are discussed later.

## Results

**Fig. 1:**
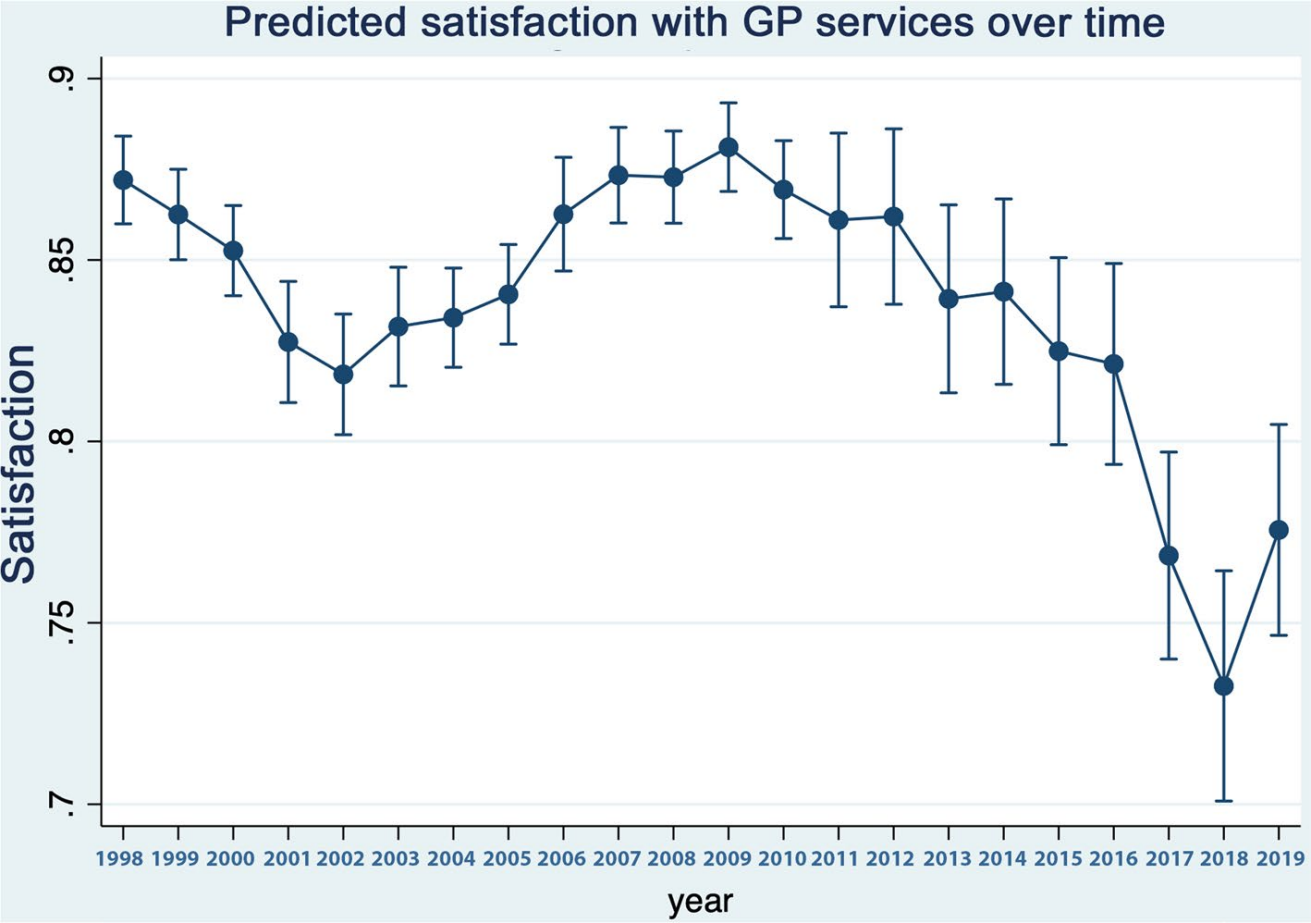
Predicted odds ratios for satisfaction, over time after controlling for covariates in the multivariable model (in Table 1)

**Fig. 2:**
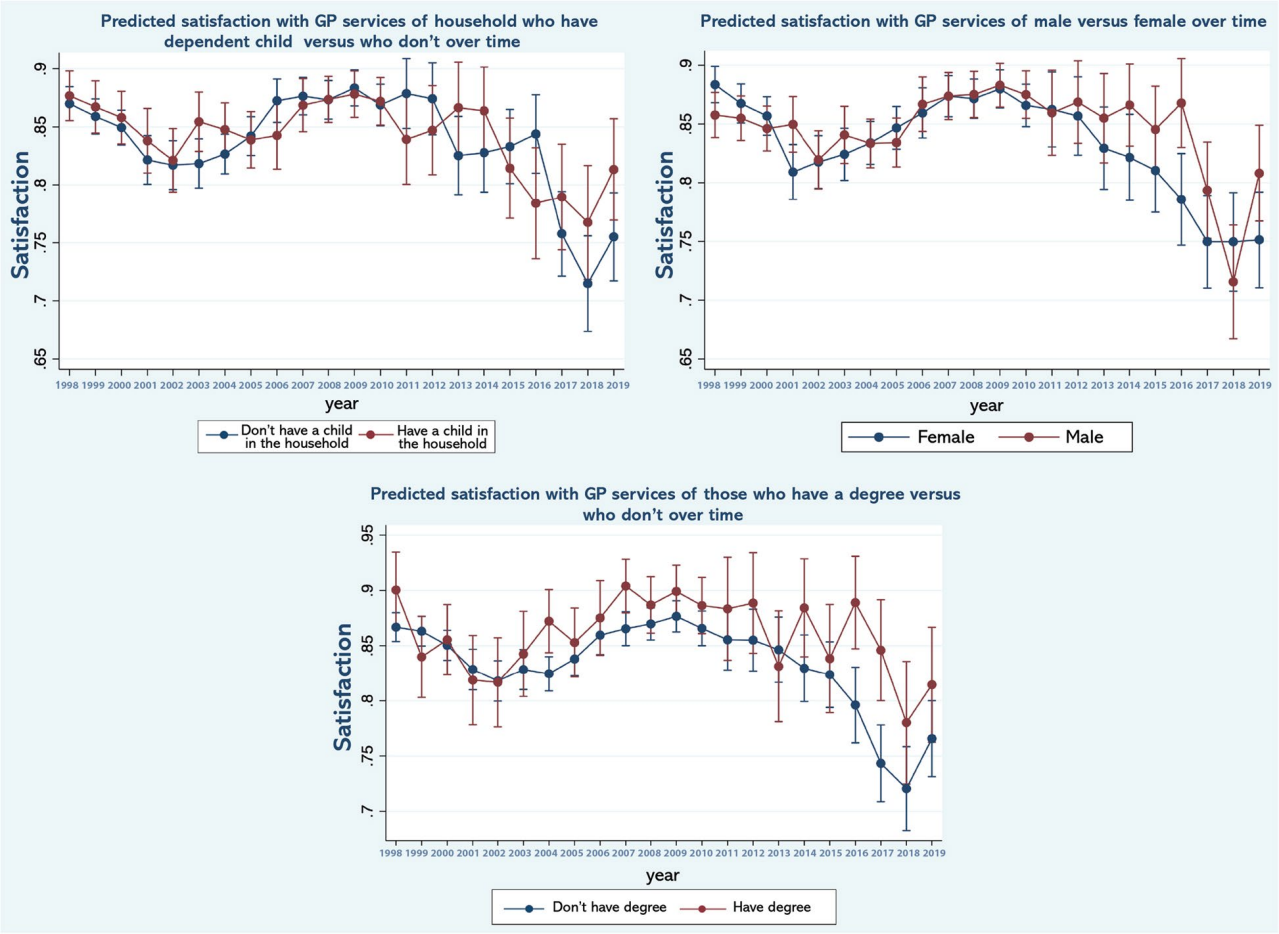
Predicted odds ratios for satisfaction, with interaction effect of time trend and other variables (i.e. having children, sex, education level)

**Fig. 3:**
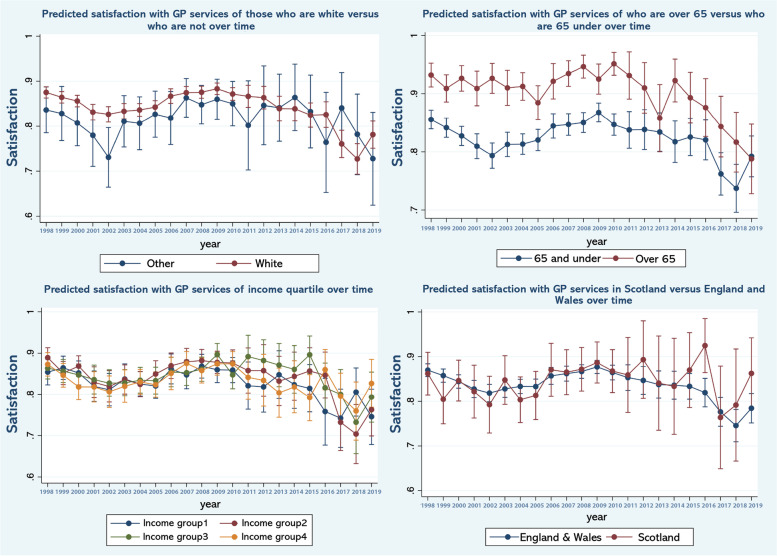
Predicted odds ratios for satisfaction, with interaction effect of time trend and other variables (i.e. race, age, income, and region)

Table 1 reports the weighted sample used as well as the percentages in various categories within it. As can be seen the usable sample size varies over the timespan of the study reflecting the numbers recruited to specific versions of the survey in different years. Table 2 reports the results of a logistic regression examining the association between satisfaction and various observable characteristics. Figure 1 shows predicted margins, that is, the predicted odds ratio for satisfaction, over time controlling for the covariates specified in Table 2. In Figs. 2 and 3, the results of the repeated analyses are presented - predicted margins are shown in models in which time trend interacts with (adjusted) variables, specifically the variables over 65 (versus younger), educated to degree or above (versus not), resident in Scotland (versus England or Wales), income quartile, dependent children in the household (versus not) male (versus female) and not-White (versus White).

**Table 1:** Sample characteristics

Population sub-group		Percentage (%)	S.D
Satisfied with GP		84.78	0.3592
Children: Yes		32.06	0.4667
Degree: Yes		18.29	0.3866
Married: Yes		55.87	0.4965
**Income level:**			
1		28.09	0.4494
2		24.80	0.4318
3		23.99	0.4270
4		23.11	0.4215
Male		44.10	0.4965
Scotland		9.18	0.2887
White		92.41	0.2647
Over 65		21.27	0.2647
**Year:**	n		
1998	2852	7.26	0.2595
1999	2896	7.37	0.2613
2000	3019	7.68	0.2664
2001	1914	4.87	0.2153
2002	1983	5.05	0.2189
2003	1951	4.96	0.2172
2004	2788	7.10	0.2568
2005	2711	6.90	0.2535
2006	1823	4.64	0.2104
2007	2394	6.09	0.2392
2008	2610	6.64	0.2491
2009	2658	6.76	0.2512
2010	2402	6.11	0.2396
2011	783	1.99	0.1397
2012	808	2.05	0.1419
2013	788	2.00	0.1402
2014	789	2.00	0.1403
2015	863	2.19	0.1466
2016	750	1.91	0.1368
2017	872	2.22	0.1473
2018	790	2.01	0.1404
2019	823	2.09	0.1432
n	39,267

**Table 2:** Multivariable logistic regression model of GP service satisfaction as a function of the variables shown

Independent variable	Odds Ratio	95% C.I	*p*-value
**Children**			
No	1		
Yes	0.980	0.9159, 1.0495	0.571
**Degree**			
No	1		
Yes	1.18	1.087, 1.291	< 0.001
**Married**			
No	1		
Yes	1.12	1.048, 1.209	0.001
**Income level**			
1	1		
2	1.09	0.9989, 1.1994	0.053
3	1.05	0.9557, 1.1594	0.297
4	0.99	0.8949, 1.0986	0.872
**Sex**			
Female	1		
Male	1.02	0.9615, 1.0906	0.459
**Region**			
Other	1		
Scotland	0.99	0.8969, 1.1111	0.977
**Race**			
White	1		
Other	1.30	1.1707, 1.4612	0.000
**Over 65**			
No	1		
Yes	2.13	1.9408, 2.3407	0.000
**Year:**			
1999	0.87	0.7400, 1.0334	0.116
2000	0.81	0.6934, 0.9613	0.015
2001	0.71	0.6010, 0.8574	0.000
2002	0.66	0.5585, 0.7918	0.000
2003	0.73	0.6128, 0.8739	0.001
2004	0.73	0.6254, 0.8668	0.000
2005	0.74	0.6268, 0.8754	0.000
2006	0.90	0.7502, 1.1027	0.335
2007	0.94	0.7860, 1.1303	0.524
2008	0.98	0.8237, 1.1713	0.842
2009	1.08	0.9047, 1.2961	0.385
2010	0.96	0.8026, 1.1546	0.682
2011	0.87	0.6822, 1.1275	0.306
2012	0.85	0.6590, 1.1173	0.256
2013	0.77	0.6051, 0.9956	0.046
2014	0.76	0.5939, 0.9845	0.037
2015	0.76	0.6077, 0.9733	0.029
2016	0.73	0.5730, 0.9338	0.012
2017	0.51	0.4148, 0.6423	0.000
2018	0.44	0.3570, 0.5582	0.000
2019	0.56	0.4517, 0.7088	0.000

Weighted sample

As can be seen in Fig. 1, satisfaction fluctuates year on year and there are clear patterns in predicted satisfaction. Thus, the period of observation begins with a fall in satisfaction that ends around 2002, followed by a period of stable satisfaction levels until 2005 and then improved levels until around 2008 followed by further sustained falls that appears to terminate around 2019. There is no obvious change in satisfaction in or immediately after 2004 that would coincide with the introduction of the Quality Outcomes Framework. Figure 2a to c shows trends in satisfaction between males and females; people with and without a university degree; and adults who have versus have not dependent children in the household. Similarly, Fig. 3a to d presents trends in satisfaction between people aged 65 and over and younger adults; different income quartiles; minority ethnic backgrounds versus White and resident in Scotland versus resident in England and Wales.

While year-on-year fluctuations are evident and there are distinct periods of falling and rising satisfaction, there does not appear to be clear, obvious differences between the sub-groups. However, Fig. 3 suggests that that there are subtle but distinct patterns. For example, the experience of income groups widens after 2008 as evident by the widening gap in predicted satisfaction between groups. By contrast, minority ethnic groups and people aged 65 and over appeared to be more likely to express satisfaction with services for much of the period under observation, with satisfaction appearing to converge with respective comparator groups after 2008.

**Fig. 4:**
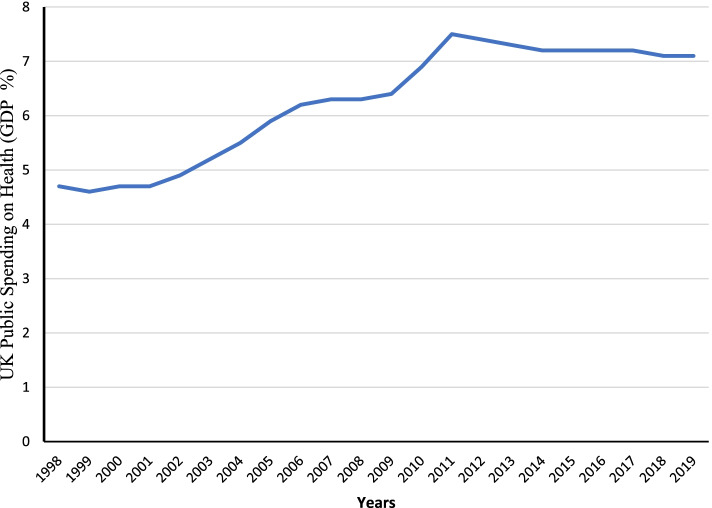
UK public health sector spending as a percentage of GDP

As can be seen in Fig. 4, using data from the Health Foundation [29], public sector spending on health as a percentage of GDP rose between 1997/98 and 2009/10 and at a faster pace between 2002 and 2006 than in the earlier period before falling up to 2018/19.

## Discussion

Within the Donabedian model of quality improvement, structure and process drive outcomes such as public satisfaction [30] The period 1998–2019 was marked by major changes to organisational structures for general practice in Britain including the abolition of fundholding and the transition from Primary Care Groups eventually to Care Commissioning Groups in England, and in Scotland and Wales greater emphasis was placed on cooperation among primary and secondary care providers. Similarly, the period was marked by significant changes to processes such as the introduction of the Quality Outcomes Framework (QOF) and arrangements around provision and delivery of out-of-hours services that were introduced as part of the new GP contract with government [31]. It unclear to what extent GP service users (especially intermittent users) were aware of such changes especially changes that were structural in nature. Arguably, public experience is influenced more immediately by factors such as perceived ease of access; and, among regular and frequent users, salient factors include the duration of consultations, the demeanour of practitioners and the speed with which onward referral results in access to secondary care. Here more fundamental though perhaps more insidious changes related in large part to funding such as staffing in primary (and secondary) care are likely to be of more immediate relevance. The quality of service delivery will suffer where there is under staffing as a result of lack of investment [32–34], growing demand related to population ageing [35], and demoralisation resulting from a lack of recognition, increased administrative demands and fear of litigation [36–41], regardless of structures and processes. While major changes in structure and process were introduced in 2003, they appear to have had little immediate impact on public satisfaction which remained static. Similarly the adoption of the Quality Outcomes in 2004 does not appear to have coincided with any uptick in satisfaction. This is perhaps understandable given the potential for inertia in a system to delay changes in provider behaviour and user experience or within a context of sustained policy change for the effects of any one policy to become dissociated from the public’s experience. By contrast the economic downturn of 2009 and the subsequent period of austerity that followed, coincided with a significant and sustained drop in public satisfaction. This finding suggests that a funding environment and all that flows from that in terms of the ability to deliver a service, especially when sustained over a prolonged period of time has greater potential to drive public satisfaction than policies which may remain quite removed from the user let alone the perceptions of the general public. That satisfaction levels correlate strongly with estimates of GPs per head of population offers support to this argument and echoes arguments of Appleby and Robertson [42].

Interestingly, perhaps, our results suggest that experience varied across specific groups of users whose circumstances may have left them more vulnerable to the prolonged period of underinvestment and growing pressure on primary care. For example, in 2010, at the outset of austerity, satisfaction levels among people aged over 65 were significantly higher than younger age groups. These levels converged over the period of austerity suggesting a sharper fall in satisfaction among older people - people whose needs and contact with services were likely to be have been greater. This is similarly, the case for those in lower income quartiles and to an extent those who were less well educated compared to those who were better educated. Equally in England and Wales where the contraction in GPs per head of population was sharper than in Scotland, satisfaction fell more sharply during the period of austerity lending further weight to the argument. That the decline in satisfaction across other groups is shared to a greater extent – for example among those with and without dependent children or related to minority status – may relate to heterogeneity within these groups that mask differences within them, for example among older persons who are also of minority status. Equally it could be simply indicative of a shared experience. These trends and differences across groups in them are worrisome within a context of population ageing, the central role of primary care in our healthcare system and the lead time required to effect change in for example GP numbers.

It is painfully evident that the service was not well positioned to cope with existing demands prior to the onset of the COVID pandemic. While practices were compelled to change during the pandemic – with a reduction in face to face consultations for example – it is difficult to imagine the extent of the pressure on the system and how much it will increase as we emerge from the pandemic and pent up demand related to neglected conditions emerges. This challenge will meet a staff whose reserves have been depleted in coping with demands during the pandemic as well as the stresses they have faced as citizens during it. Relationships between the public and the care providers are likely to become strained for a time a least. Calls for sustained and significant increases in funding to health and social care have been met with a proposed hypothecated tax in the UK [43], a measure that is by no means a panacea. While issues regarding the fairness of the tax exist, few will argue of the need for additional resources and the importance of these resources finding their way to frontline services in a sustained manner. This is especially so given the lead time required to train a GP [34]. Other fundamental changes to GP services have been proposed and argued [31] that may be embodied in a raft of future policy initiatives. Fundamentally though, our analysis echoes the findings of L’Esperance et al. [24] and suggests significant and sustained investment in services will be required to improve patient or service user satisfaction and, in turn, the health and well-being benefits that ensue.

## Limitations

There are a number of limitations to our analysis. Firstly, while the data allowed us to capture aspects of respondents’ socio-demographic characteristics, we lacked other indicators. For example, we have no data about respondent health status – other perhaps than how this is correlated with age – and thus no real insight into their primary healthcare needs. Similarly, we have no information about their use of GP services or when service utilisation occurrred. While we are able to distinguish between ethnic groups in broad terms (White versus Non-white) that over 92% of the sample were White means it was not possible to explore heterogeneity among the Non-white minority. How such factors may have helped frame respondent expectations, experience and satisfaction are unknown, though they are likely to have done so. Second, the data are cross sectional in nature and the relationships described should in consequence be interpreted as associations rather than causal. While we are able to say for example, that older people were more likely to be satisfied than younger people we cannot with this data say how satisfaction among respondents would change as they age. In both instances these are limitations imposed on us by the data and are areas that warrant further research. Third, assessments of satisfaction may anticipate or lag behind the enactment of policies to change structure, process or resourcing rather than necessarily being contemporaneous with them. New policies may reinforce, undermine or even reverse those which precede them. The frequency and rapidity of change during this period would make it difficult to establish as clear break in the time series that would allow the effect of a specific reform to be examined with this data as is the case with funding.

## Conclusions

The period 1998–2019 witnessed a series reforms to the structure and processes of general practice across the UK. These had the potential to change the quality of care, the user’s experience and public satisfaction with services. Relating policy to satisfaction is challenging for a variety of reasons that include the volume of policies enacted over this period. Our analysis shows an association between satisfaction during a period of policy churn and distinct funding environments. It suggests that while policies change, satisfaction moves with levels of investment in a clear and intuitive manner. As the NHS emerges from the COVID pandemic, facing potentially significant pent up demand and competing demands for public resources, it is important to restate the importance of general practice to the overall success of the health service and of sustained investment in those services to public satisfaction with them.

## Supplementary Information

**Additional file 1.**

## Data Availability

Summary data is available from corresponding author upon reasonable request.

## Abbreviations

UK: United Kingdom
GP: General Practice
BSAS: British Social Attuited Survey
COVID: Corona Virus Disease
GDP: Gross Domestic Product
NHS: National Health Services
