# Supplementary figures and images for: Changes in public satisfaction with GP services in Britain between 1998 and 2019: a repeated cross-sectional analysis of attitudinal data
Source: BMC Prim Care. 2022 Apr 18;23:83. doi: 10.1186/s12875-022-01696-w (PMC9014779; doi:10.1186/s12875-022-01696-w)

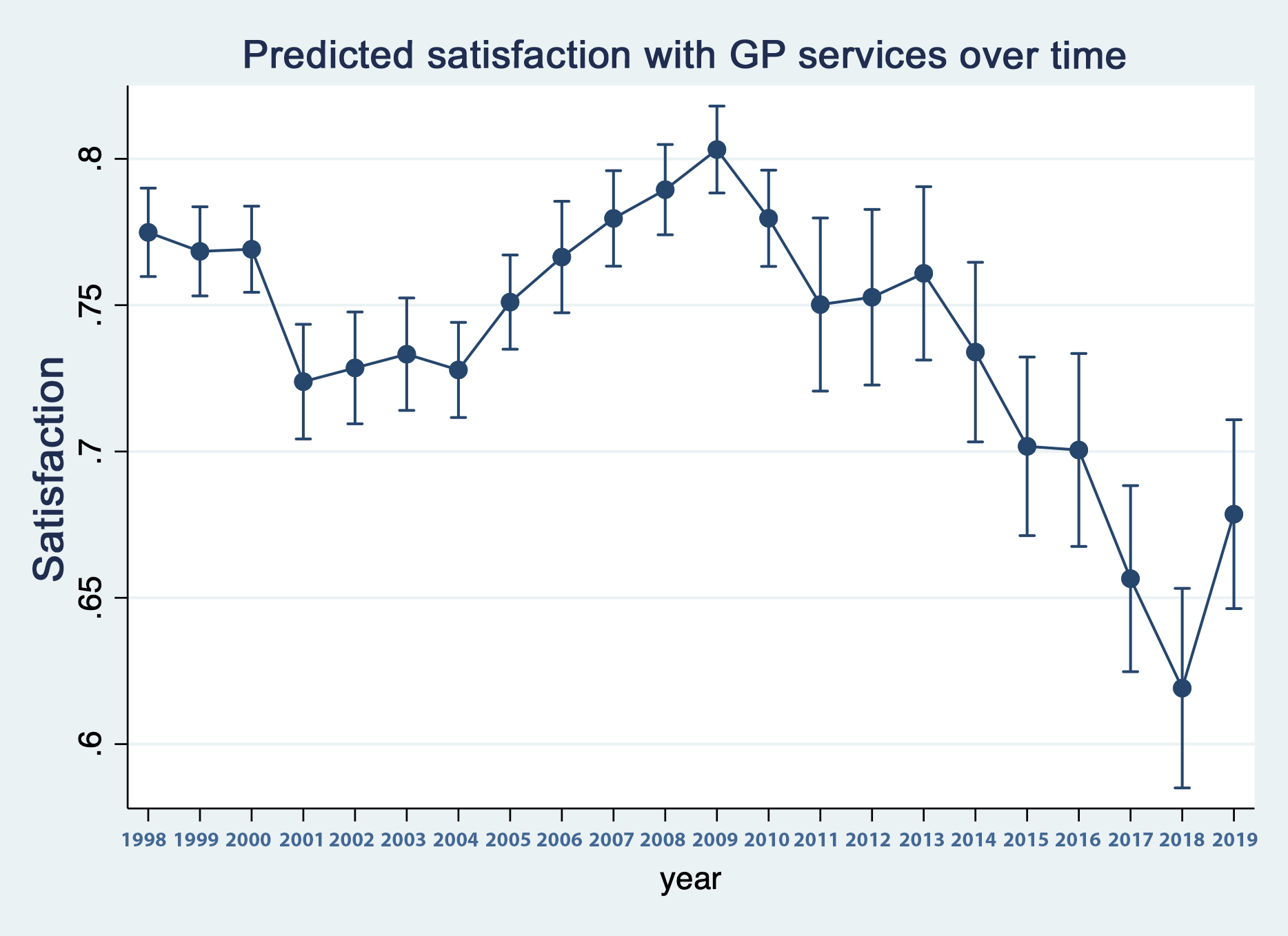

Supplement: Supplementary file 1 — Additional file 1. [file 12875_2022_1696_MOESM1_ESM.jpg]
